# Proliferative Kidney Disease and Viral Pathogens in Wild Brown Trout (*Salmo trutta*) Populations in the Czech Republic

**DOI:** 10.1111/jfd.70158

**Published:** 2026-03-07

**Authors:** Miroslava Palíková, Ivana Mikulíková, Tomáš Doležal, Ivana Papežíková, Kateřina Matějíčková, Jitka Motlová, Hana Novotná, Ivona Toulová, Jan Grmela, Anna Šindelářová, Ľubomír Pojezdal

**Affiliations:** ^1^ Department of Ecology and Diseases of Zoo Animals, Game, Fish and Bees, Faculty of Veterinary Hygiene and Ecology University of Veterinary Sciences Brno Brno Czech Republic; ^2^ Department of Zoology, Fisheries and Hydrobiology, Faculty of AgriSciences Mendel University in Brno Brno Czech Republic; ^3^ Department of Infectious Diseases and Preventive Medicine Veterinary Research Institute Brno Czech Republic

**Keywords:** climate change, PRV‐3, *Tetracapsuloides bryosalmonae*, wild salmonid populations

## Abstract

Populations of wild brown trout ( *Salmo trutta* ) have been in long‐term decline across Central Europe, including the Czech Republic, with multiple factors, such as habitat alteration, climate change, predation and emerging diseases, implicated. Among the diseases, proliferative kidney disease (PKD), caused by the myxozoan *Tetracapsuloides bryosalmonae*, has gained increasing attention. Between 2020 and 2024, we investigated the occurrence of *T. bryosalmonae* and selected viral pathogens in wild brown trout populations from 34 streams (51 sites) across the three major Czech river basins (Elbe, Morava, Odra). In total, 501 fish were examined using pathology and molecular techniques. While *T. bryosalmonae* DNA was detected in 54.7% of fish and at 74.5% of localities, with highest prevalence in the Elbe basin (63.5%), gross kidney changes consistent with PKD were observed in just 7.4% of individuals. Significant associations were found between parasite occurrence and mean summer water temperature, with positive sites showing higher water temperatures. No mortalities were observed in the wild populations, though subclinical infections were common. Our findings demonstrate that not only is the PKD agent widespread in Czech trout populations but that temperature plays an important role in parasite dynamics, highlighting increasing risks posed by climate change. We suggest that careful fish stocking strategies will be essential in preventing further spread. The viral pathogens *Salmonid novirhabdovirus, Piscine novirhabdovirus and Aquabirnavirus salmonidae* were not detected, and piscine orthoreovirus genotype 3 was confirmed in two samples from the Odra basin, showing high sequence identity with previously reported Czech isolates.

## 1 Introduction

In the Czech Republic, populations of native salmonid species, specifically brown trout ( *Salmo trutta* ) and European grayling ( *Thymallus thymallus* ), have experienced a general decline over the past 30 years, as evidenced by long‐term catch statistics from Czech fisheries (Lyach and Remr 2020; Avramović et al. 2024; Šlapanský et al. 2024). This trend, which is not limited to the Czech Republic but observed across most European countries, has been driven by multiple interacting factors, including altered hydrological and thermal regimes (Li et al. 1994; Webb et al. 2003; Donadi et al. 2023; Leach et al. 2023), morphological modifications to stream channels (Letcher et al. 2007; Tamario et al. 2021; Lyach 2022), increased pressure from piscivorous predators (Jacobsen 2005; Chalupa et al. 2016; Zapletal et al. 2021) and the emergence of salmonid‐specific diseases. Recently, the impact of proliferative kidney disease (PKD) on salmonids has received increasing attention, with high prevalences reported from numerous European countries (e.g., Hedrick et al. 1993; Waldner et al. 2019; Ros et al. 2021; Lauringson et al. 2021).

*Tetracapsuloides bryosalmonae*, a myxozoan endoparasite, is the causative agent of PKD, an emerging parasitic infection affecting freshwater salmonids across Europe and North America (Okamura et al. 2011; Sudhagar et al. 2019). The disease causes high mortality in both wild and farmed populations (Schmidt‐Posthaus et al. 2015; Gorgoglione et al. 2016; Lewisch et al. 2018; Waldner et al. 2019), with infection prevalence in aquacultural facilities reaching 100% and mortality ranging from 15% in subclinical cases to 95%–100% during outbreaks exacerbated by secondary infections or environmental stressors (Bailey et al. 2017; Schmidt‐Posthaus et al. 2012, 2015; Gorgoglione et al. 2016). Two genetically distinct *T. bryosalmonae* strains have been identified, one from North America and the second from Europe (Henderson and Okamura 2004). The agent's life cycle includes different species of freshwater bryozoan as invertebrate hosts and salmonids as vertebrate hosts. The transmission of the parasite and PKD development are both strongly temperature‐dependent (Hedrick et al. 1993). Temperature is a crucial factor influencing PKD outbreaks. It directly affects not only the parasite by regulating its proliferation and development, but also both hosts. Elevated water temperatures enhance primary production, thereby supporting the development of bryozoan colonies that produce malacospores infective for salmonids, and simultaneously lead to more severe kidney hyperplasia in infected fish (Okamura et al. 2011). Conversely, under otherwise favourable environmental conditions, *T. bryosalmonae* infection does not result in PKD when summer water temperatures remain below 15°C (Ros et al. 2021; Wahli et al. 2002). Consequently, ongoing warming of riverine environments is expected to further enhance the impact of PKD on salmonid populations (Borgwardt et al. 2020).

In the Czech Republic, overt mortalities of salmonids in open waters due to PKD have not yet been demonstrated. However, it should be noted that it is virtually impossible to monitor and demonstrate fish mortalities in open waters unless they occur on a large scale (Pojezdal et al. 2020). On the other hand, serious losses have been reported in rainbow trout ( *Oncorhynchus mykiss* ) reared in recirculating aquacultural systems (Palíková et al. 2017). Recent PKD outbreaks in Norway have demonstrated the potential of the disease to cause mass mortalities in wild salmonids (Sterud et al. 2007; Mo and Jørgensen 2016). The disease has also been linked to long‐term declines in Swiss brown trout populations (Rubin et al. 2019). The presence of the PKD agent, *T. bryosalmonae*, has been confirmed in wild salmonid species such as Atlantic salmon ( *Salmo salar* ), brown trout, brook trout ( *Salvelinus fontinalis* ), Arctic char ( *Salvelinus alpinus* ) and European grayling from many European countries, including Austria, Finland, Denmark, Slovenia, Estonia and even Iceland (Wahli et al. 2002; Kristmundsson et al. 2010, 2023; Skovgaard and Buchmann 2012; Jenčič et al. 2013; Dash and Vasemägi 2014; Schmidt‐Posthaus et al. 2015; Vasemägi et al. 2017; Waldner et al. 2019; Ros et al. 2022) and has been found parasitising European whitefish ( *Coregonus lavaretus* ) in Finland and Norway (Sobociński et al. 2018; Oredalen et al. 2021). The American strain of *T. bryosalmonae* was also detected in mountain whitefish ( *Prosopium williamsoni* ) during a severe PKD outbreak with high mortality in the Yellowstone River (Hutchins et al. 2021). In recent years, PKD monitoring has been undertaken on wild brown trout populations in the Czech Republic to assess whether the disease may be one of the possible causes of salmonid declines in open waters.

The Czech Republic is not declared free of notifiable diseases of salmonids (except for Infectious salmon anaemia) and presence of *Piscine novirhabdovirus* and *Salmonid novirhabdovirus* manifesting as viral haemorrhagic septicaemia (VHS) and infectious haematopoietic necrosis (IHN), respectively, is confirmed in occasional, but regular, outbreaks in farmed salmonids. Presence of *Aquabirnavirus salmonidae*, previously infectious pancreatic necrosis virus (IPNV), was confirmed in the Czech Republic, but is most probably underreported due to lack of regulation considering the disease (Pojezdal et al. 2017). Piscine orthoreovirus 3 (PRV‐3) has been shown to be a pathogen of rainbow trout farmed in intensive conditions, but its impact on the health of brown trout, especially outside of the farming conditions, needs to be examined (Olsen et al. 2015). The presence of the virus has previously been confirmed in the Czech population of wild brown trout (Pojezdal et al. 2020), along with other countries in continental Europe (Dhamotharan et al. 2018).

This paper aims to present evidence on the distribution of *T. bryosalmonae* and selected viral agents in the main salmonid streams in the Czech Republic, provide new insights into the *T. bryosalmonae* issue in the field (correlation between *T. bryosalmonae* prevalence and mean summer water temperatures) and propose new stocking strategies preventing further spread of *T. bryosalmonae*.

## 2 Material and Methods

### 2.1 Fish Sampling

Fish (brown trout only) were obtained by electrofishing between July and September (except for one sampling in November) in 2020–2024, with an average of 10 (2–17) fish sampled per locality. In total, 501 fish were sampled from 51 localities on 34 streams. The GPS coordinates of the sampling localities are provided in Supporting Information. S1. Fish were measured and their age was estimated based on total length, according to the length–frequency distribution of the population at each locality. Age class 1+ was primarily selected; if this age group was not present at a given locality, fish of age classes 0+ or 2–3+ were sampled instead.

Fish were first euthanised by a blow to the head followed by cutting the vertebral column and vessels at the base of the skull, a procedure in accordance with national legislation, specifically Act No. 246/1992 Coll., on the Protection of Animals against Cruelty, as amended. The fish were then examined on site for the presence of pathoanatomical signs of PKD, with an emphasis on changes to the kidneys. During autopsy, spleen, heart, and cranial kidney tissue samples were preserved in 70% ethanol for virological examination. In addition, caudal kidney tissue was also fixed in 70% ethanol for PCR detection of *T. bryosalmonae*.

### 2.2 Temperature Measurement

Data supplied by the Czech Hydrometeorological Institute were used alongside temperatures measured on site at the time of sampling to evaluate temperature differences between localities. Where a Czech Hydrometeorological Institute limnigraph was located at or near the fish sampling site, the number of days with a mean daily temperature > 15°C was calculated from the beginning of the year up to the date of fish sampling. The mean daily temperature was calculated from measurements recorded at 10‐minute intervals. Moreover, the mean summer temperature (July–August) of the year of capture was calculated in order to evaluate the temperature regime of the monitored streams.

If there was no limnigraph nearby, temperatures were measured on site at the time of the sampling using a Hach HQ40D multimeter (Hach, Germany).

### 2.3 Parasitological Examination

Presence of kidney swelling was evaluated as either 0 (not present) or 1 (present). Individual samples of kidney tissue fixed in 70% ethanol were used for molecular detection of *T. bryosalmonae* 18S DNA using the real‐time PCR assay. Total DNA for the analysis was extracted using the E.Z.N.A. Tissue DNA kit (Omega, Bio‐Tek, USA). The real‐time PCR was prepared according to Bettge et al. (2009), briefly: Each 10 μL reaction contained 5 μL of TaqMan Universal PCR Master Mix (Applied Biosystems, USA), 0.3 μM primers PKDtaqf1 (5′‐GCGAGATTTGTTGCATTTAAAAAG‐3′) and PKDtaqr1 (5′‐CACATGCAGTGTCCAATCG‐3′), 0.2 μM fluorescent probe probePKD (5′‐FAM‐CAAAATTGTGGAACCGTCCGACTACGA‐TAMRA‐3′) and 2.5 μL of DNA extract. Amplification was done in the qTOWER^3^ PCR system (Analytik Jena, Germany) with the following amplification conditions: 50°C for 2 min and 95°C for 10 min, followed by 45 cycles of 95°C for 15 s and 60°C for 1 min. Each PCR plate contained a positive control and a negative (non‐template) control.

### 2.4 Virological Examination, Phylogenetic Analysis

Presence of specific viral salmonid pathogens was assessed using established conventional and real‐time PCR assays targeting *Piscine novirhabdovirus* (VHSV), *Salmonid novirhabdovirus* (IHNV), IPNV and PRV‐3 RNA (Pojezdal et al. 2020). Briefly, internal organs from five fish were pooled, mechanically lysed, suspended in a 10‐fold volume of cell culture medium and centrifuged. The supernatant was used directly in PCR assays following nucleic acid extraction. All positive samples were analysed via Sanger sequencing performed by SeqMe (Dobříš, Czechia). Nucleotide sequences of the PRV‐3 segment S1 were compared to piscine orthoreovirus sequences available in GenBank using BLAST (Altschul et al. 1990) and aligned using BioEdit v. 7.2.5. (Hall 1999).

### 2.5 Statistical Evaluation

Real‐time PCR *T. bryosalmonae* data were statistically analysed using contingency tables and chi‐square tests. Where the Shapiro–Wilk test revealed a non‐normal data distribution, Spearman's rank correlation coefficient, which allows the degree and direction of dependence between two variables to be assessed, was used to evaluate the relationship between mean summer temperature and *T. bryosalmonae* prevalence. During data processing, three outlying values were excluded on the basis that (a) *T. bryosalmonae* positivity appeared to be independent of temperature, and (b) the fish positive for *T. bryosalmonae* DNA were most likely stocked at the three localities (Jizerka, Lučina, Kamenice). Differences in the frequency of pathological changes in PCR positive fish among individual age categories of fish were evaluated using the Pearson chi‐square test. Subsequent pairwise comparisons between groups were conducted using 2 × 2 contingency tables with Yates' continuity correction. The association between *T. bryosalmonae* occurrence in fish and sampling time was assessed using Fisher's exact test. All statistical tests were evaluated at a significance level of *p* < 0.05. All analyses were performed using Unistat software for Excel v. 6.5.

## 3 Results

Enlarged kidneys were present in 37 specimens (7.4% of fish examined), with 10–100% prevalence per location. All fish showing kidney hyperplasia were also positive for *T. bryosalmonae* DNA using real‐time PCR. No other pathological changes were observed in the fish examined. While kidney pathology was only detected in a relatively low number of fish, real‐time PCR revealed the presence of *T. bryosalmonae* DNA in 274 fish (54.7% of all fish examined) from 38 localities (74.5%), only 13 localities proving negative.

**FIGURE 1:**
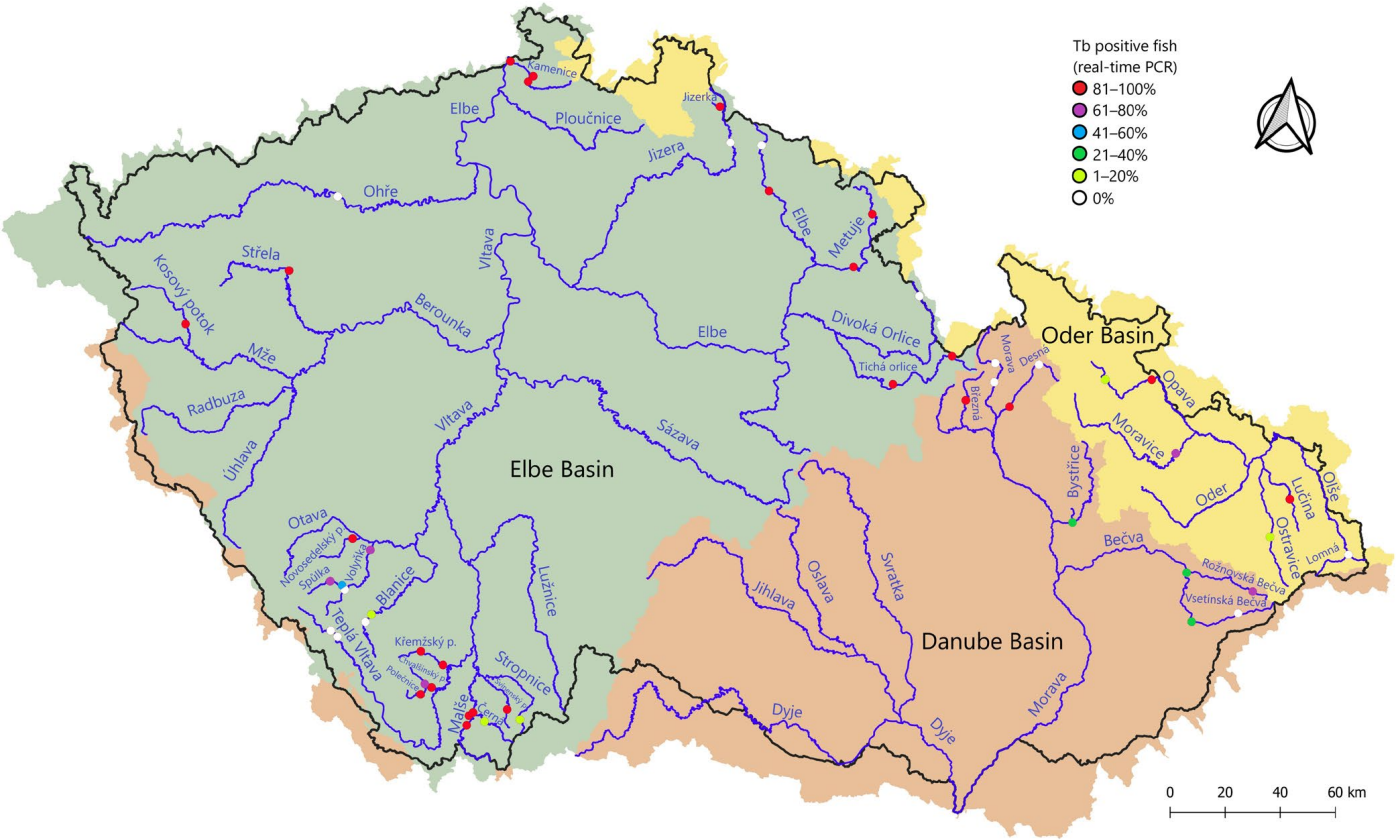
*T. bryosalmonae* prevalence at the localities examined in the Czech Republic.

**FIGURE 2:**
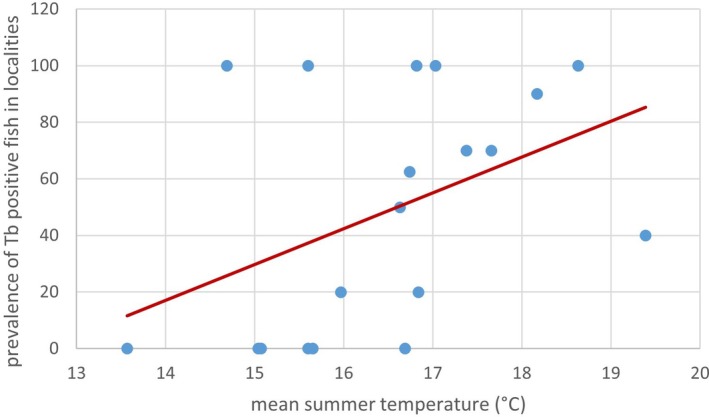
Dependence of *T. bryosalmonae* DNA presence on the number of days with mean summer temperatures > 15°C. Localities Jizerka, Lučina and Kamenice are not included in the correlation.

While no fish mortalities were observed in any of the streams monitored, the Elbe basin showed a particularly high prevalence of *T. bryosalmonae* DNA positive fish (*n* = 209, 63.5%) with 27 of 35 localities proving positive (77.1%), while 41 fish (40.2%) from six localities (60.0%) were positive in the Morava basin, and 24 fish (34.3%) from five of the six sites monitored (83.3%) in the Oder basin (Supporting Information S1 and Figure 1). Though numbers of positive fish were significantly higher in the Elbe basin compared with the Oder and Morava basins (*p* < 0.001), there was no significant difference in numbers of positive localities between basins. There was a positive correlation between occurrence of *T. bryosalmonae* and number of mean summer temperatures (*p* < 0.05; Figure 2). The highest percentage of kidney pathology in PCR‐positive fish was detected in the 0+ fish age class (85%), this category shows a highly significant difference compared with age categories 1+, 2+ and 3+ (*p* < 0.0001), whereas no significant differences were detected among the remaining groups (Table 1). No dependence on sampling time (i.e., differences between months) was revealed.

**TABLE 1:** Presence of pathological changes typical for PKD (enlarged kidney) in individual age categories of PCR‐positive fish for *Tetracapsuloides bryosalmonae*.

Fish/age	0+	1+	2+	3+
PCR positive fish	20	101	133	20
Pathological changes present	17	6	12	2
Pathological changes absent	3	95	121	18
% of positive fish	85.0	5.9	9.0	10.0

*Note:* Numbers of 0+ fish with presence of pathological changes significantly differed from all other categories (*p* < 0.0001).

While PCR assays targeting VHSV, IHNV, and IPNV proved negative for all samples, two samples were positive for PRV‐3. In both cases, the fish came from the Odra basin (streams Moravice and Ostravice, CZ‐2543 and CZ‐2549, respectively). All samples from the Elbe and Morava basins proved negative (Supporting Information S2). BLAST analysis of the 371 bp nucleotide sequences from the PRV‐3 positive samples found 19 published PRV‐3 sequences with 100% identity to the CZ‐2543 isolate (ref. no. PX216780). Of the Czech isolates, only the previously published Danube basin isolates (CZ‐1964/1965, ref. no. MT572468) were found to be 100% identical with each other. Danube basin isolates and the current Moravice CZ‐2543 and Ostravice CZ‐2549 isolates (ref. no. PX16781) all differed by a single nucleotide.

## 4 Discussion

In wild brown trout populations, *T. bryosalmonae* can only spread through the distribution of its main hosts, *Fredericella* spp. and/or *Plumatella* spp. (Anderson et al. 1999; Longshaw et al. 1999; Okamura and Wood 2002). While we observed a high overall prevalence of the causative agent of PKD in Czech brown trout, with just a few exceptions, fish caught at higher elevations, close to stream headwaters, were usually *T. bryosalmonae* negative. This finding is consistent with previous studies reporting *T. bryosalmonae* as predominantly occurring in the lower reaches of rivers, with the upper reaches typically free of infection (see Bruneaux et al. 2016; Rubin et al. 2019; Schmidt‐Posthaus et al. 2021). At higher elevations, where water temperatures remain low throughout the year, bryozoan occurrence is markedly reduced, suggesting that such conditions are generally unfavourable for bryozoan establishment (Økland et al. 2003). In addition to suboptimal thermal regimes, such sites have higher flow velocities and lower organic matter content, resulting in limited food availability for filter feeders such as Bryozoa (Hartikainen et al. 2009). Similarly, Ros et al. (2021), in a study focusing in part on the predictive modelling of bryozoan distribution, reported that both higher elevation sites (specifically the Black Forest and north‐eastern Baden‐Württemberg) and lowland river stretches (Rivers Rhine and Neckar, Germany) provided unsuitable habitat for the Bryozoa *Fredericella sultana* and *Plumatella emarginata* . Their presence was found to correlate positively with temperature seasonality, characterised by summer and winter air temperature peaks of approximately 17°C and 0°C, respectively.

In our study, *T. bryosalmonae* was detected on a single occasion at an upstream site located above a downstream locality where the disease was recorded as absent. *T. bryosalmonae* was detected on the River Jizerka, the highest altitude site in the study with the lowest mean summer temperature. Samples taken downstream of the Jizera were negative, despite it being a receiving watercourse for the Jizerka. The Jizerka is also characterised by low water temperatures throughout the year, with the Czech Hydrometeorological Institute reporting no days when the temperature exceeded 15°C in the year of sampling (Supporting Information S1). We hypothesise that *T. bryosalmonae* was introduced to this area via stocked fish. Elucidating these relationships will be the focus of future studies (fish sampling of farms producing stock salmonid fish for selected localities). The low temperature of the water may explain why the agent has not yet spread downstream, given that bryozoan spores are released in greater numbers at higher temperatures (Tops et al. 2006). Furthermore, impassable weirs separate the trout populations along this river, effectively reducing downstream migration and preventing upstream migration. Likewise, we suppose that infected fish were introduced at the Lučina and Kamenice localities, where water temperatures had not exceeded 15°C until the day of fish capture in the sampling year and the mean summer temperature was 13.28°C and 12.35°C, respectively. According to Rubin et al. 2019, the number of days per year with temperatures above 15°C is a key factor in the development of the disease, presence of a suitable invertebrate host and subsequent release of infectious spores into the aquatic environment.

No severe mortalities were reported from the Czech localities unlike other European studies (e.g., Sterud et al. 2007) and only a small proportion of the trout examined showed pathological changes corresponding to PKD. Enlarged kidneys or spleen were recorded in just 7.4% of the fish examined, all of which came from 10 localities in the Elbe basin, representing 19.6% fish in total. In most cases, prevalence was low, with enlarged kidneys found in just one or two fish at each locality (10%–25% prevalence). A prevalence greater than 40% was only recorded at three localities, that is, Březná, Srbská Kamenice and Malše. Overall, the prevalence of kidney hyperplasia in fish that tested positive for *T. bryosalmonae* DNA reached 13.5%. Ros et al. (2021) described a substantially higher percentage of kidney hyperplasia in brown trout testing positive (57.9%; prevalence 60.7%), and also recorded a higher rate of kidney damage in brown trout compared to Atlantic salmon and grayling. Note, however, that the proportion of positive localities in our own study (74.5%) was similar to that reported by Ros et al. (2021) (78.5%). The highest percentage of pathological changes was detected in the 0+ fish PCR‐positive fish; however, this group was the least represented among the sampled fish overall. Fish of the 0+ age class were collected at only three localities, and pathological changes were detected in fish from only one of these three sites. At the locality where three fish age classes were represented, the occurrence of pathoanatomical changes was comparable among age groups. The correlation between positive localities and their water temperature confirms the key role of the latter for occurrence of *T. bryosalmonae*.

PCR frequently detects *T. bryosalmonae* in salmonids without accompanying histopathological kidney lesions, indicating latent or subclinical infections (Skovgaard and Buchmann 2012; Schmidt‐Posthaus et al. 2015). Our results are consistent with this pattern, as PCR identified positive sites in the absence of detectable pathology. This highlights the higher sensitivity of molecular methods and suggests that reliance solely on histopathology may underestimate *T. bryosalmonae* prevalence, particularly under cold‐water conditions or at early stages of infection.

Indeed, the development of detectable histopathological changes is strongly temperature‐dependent, with experimental studies showing that a cumulative mean of approximately 1500 degree‐days, or at least 30 consecutive days with a daily mean temperature ≥ 15°C, is required before infection can be consistently demonstrated histologically (Rubin et al. 2022). According to Ros et al. (2021), *T. bryosalmonae*‐free headwaters will likely play a major role in the survival of young salmonids in the context of climate change. As such, these populations should be carefully protected from PKD infection.

The results gathered in this study should inform the assessment of future salmonid stocking strategies in the Czech Republic. Since brown trout are active hosts of *T. bryosalmonae* and can release infectious stages into the water, infected fish should not be stocked in areas where bryozoans are present and where no *T. bryosalmonae* DNA has yet been detected, or where bryozoans are likely to spread in future due to climate change. Also, lower stretches should be stocked by *T. bryosalmonae*‐free fish sources due to brown trout upstream migration, although for example Schmidt‐Posthaus et al. (2021) did not observe *T. bryosalmonae* spreading from lower *T. bryosalmonae* positive parts to upper *T. bryosalmonae* negative parts by migrating brown trout. This measure should prevent environmental contamination and transmission to naive wild trout populations. Such localities should be protected primarily by stocking fish from *T. bryosalmonae*‐free farms. Furthermore, brown trout can excrete infectious spores into the environment via their urine for a prolonged period, making them significant carriers. Indeed, studies have shown that such fish can be chronic carriers, excreting spores into the water for at least 2 years after initial exposure (Abd‐Elfattah et al. 2014; Soliman et al. 2018). Localities where infected fish are believed to have been stocked, but where the parasite's life cycle probably has not yet been completed due to the presumed absence of an invertebrate host and current low water temperature should also be fished out and restocked with uninfected trout, with future stocks coming from *T. bryosalmonae*‐free sources. If there are no bryozoans present at the locality (the application of eDNA analyses may provide greater confidence), infected trout should disappear from the site within a few years due to their relatively short lifespan. A similar approach could be taken for localities in the upper reaches of rivers and those with cold water where the mean summer water temperature does not exceed 15°C (see Figure 2). Conversely, streams where summer water temperatures consistently exceed 15°C and where the presence of parasite DNA has been detected should be considered contaminated. In such cases, it is likely that occurrence of *T. bryosalmonae* is not limited to stocked fish and that the pathogen is present in the environment, completing its life cycle. This assumption would be confirmed by finding infected bryozoa.

Presence of PRV‐3 was first detected in the Czech Republic in two rivers in the Danube basin (Pojezdal et al. 2020). This study has now expanded the known range of this virus by confirming its presence in wild brown trout in two rivers in the Odra basin. However, unlike the intensively farmed rainbow trout examined by Olsen et al. (2015), none of the most recent positive fish showed clinical or pathoanatomical signs of PRV‐3 infection. It remains unclear whether this is due to differences in susceptibility between brown and rainbow trout, or whether environmental factors such as water temperature may be involved (Sørensen et al. 2023). In either case, further studies are needed to properly assess the virus's impact on wild brown trout populations. Rainbow trout is the most susceptible species to the viral diseases monitored. Species differences may also have been a factor explaining the absence of VHSV, IHNV and IPNV in the brown trout examined in this study. Nevertheless, a few VHS and IHN outbreaks are recorded in the Czech Republic each year (Pojezdal et al. 2017), with trade in live fish or eggs considered the primary vector across Central Europe (Reichert et al. 2013). Although water temperatures usually preclude natural propagation via rivers, temperatures in our study were suitable for viral propagation in at least 11 rivers at the time of sampling; therefore, natural propagation cannot be ruled out.

Data for the 371 bp nucleotide sequence encoding the S1 segment of the sigma 3 protein suggest that the genetic makeup of the PRV‐3 is highly conservative. The BLAST analysis of the GenBank database revealed 19 PRV‐3 isolates that were 100% identical to the CZ‐2543 isolate (ref. no. PX216780), which was found to have a wide temporal (1995–2024) and geographical distribution (Poland, Chile, Denmark, Peru, Germany and Italy). Even under these conditions, the Czech isolates demonstrated some variability, with only the Danube basin isolates (CZ‐1964/1965, ref. no. MT572468) 100% identical, and two of the Czech isolates (CZ‐1964/1965 and CZ‐2549) having no 100% matches in the database. Further study of a larger sequence, alongside isolation of samples from additional countries, will be required to draw definite conclusions on the potential origin and dispersion of the PRV‐3 virus.

## 5 Conclusion

The present study demonstrated that *T. bryosalmonae*, the agent of proliferative kidney disease, is widespread among wild brown trout populations across the main Czech river basins. Significant correlations were revealed between parasite occurrence and the numbers of days with mean summer water temperatures above 15°C. The highest prevalence of *T. bryosalmonae* was recorded in the Elbe basin. Although gross kidney alterations consistent with PKD were only detected in a small proportion of fish, molecular analysis revealed frequent subclinical infections, indicating that the parasite is broadly distributed in the environment. The viral pathogens VHSV, IHNV, and IPNV were not detected, and PRV‐3 was confirmed in just two samples from the Odra basin. These findings, although limited in scope, suggest that parasitic agents currently pose a greater health threat to wild brown trout populations in the Czech Republic than the viral agents examined. With increasing river temperatures linked to climate change, further expansion of *T. bryosalmonae* may be expected. Consequently, careful management of stocking practices using *T. bryosalmonae*‐free fish sources and the protection of cold‐water refugia will be essential to prevent further spread and to maintain viable wild trout populations.

## Author Contributions

M.P.: Conceptualization, investigation, visualisation, supervision, writing – review and editing, writing – original draft, funding acquisition, methodology, data curation. I.M.: Writing – original draft, writing – review and editing, investigation, project administration, validation. T.D.: Investigation, methodology, writing – original draft, formal analysis, resources, conceptualization. I.P.: Investigation, writing – review and editing, visualisation. K.M.: Investigation, formal analysis. J.M.: Investigation, formal analysis. H.N.: Investigation, formal analysis. I.T.: Investigation. J.G.: Conceptualization, methodology, funding acquisition, writing – review and editing. A.Š.: Methodology, investigation. Ľ.P.: Writing – original draft, resources, data curation, validation, writing – review and editing.

## Funding

This work was supported by the Ministry of Agriculture of the Czech Republic (Project QK23020064) and by project FVHE/Pikula/2025ITA22 and European Regional Development Fund in the Operational Programme Research, Development and Education and The Czech Ministry of Education, Youth and Sports, project PROFISH [CZ.02.1.01/0.0/0.0/16_019/0000869].

## Conflicts of Interest

The authors declare no conflicts of interest.

## Supporting information


**Supporting Information: S1.** Characteristics of sampling sites and sampled fish, prevalence of kidney pathology and *T. bryosalmonae* positive real‐time PCR at each locality.

**Data S1:** jfd70158‐sup‐0002‐Supinfo.docx.

## Data Availability

The data that support the findings of this study are available from the corresponding author upon reasonable request.
