# Supplementary material for: Proliferative Kidney Disease and Viral Pathogens in Wild Brown Trout (Salmo trutta) Populations in the Czech Republic
Source: J Fish Dis. 2026 Mar 7;49(8):e70158. doi: 10.1111/jfd.70158 (PMC13331547; doi:10.1111/jfd.70158)
Supplement: Supplementary file 2 — Data S1: jfd70158‐sup‐0002‐Supinfo.docx. [file JFD-49-e70158-s001.docx]

Suppl. 1. Characteristics of sampling sites and sampled fish, prevalence of kidney pathology and *Tetracapsuloides bryosalmonae* (Tb) positive real-time PCR at each locality (N = number of fish examined, TL = total length, T = temperature at time of sampling, CHMI = Czech Hydrometeorological Institute, n.a. = not available, SY = sampled year). ^1^localities of Oder basin, ^2^localities of Morava basin, ^3^localities of Elbe basin

| **Date** | **River** | **GPS** | **N** | **TL (mm) /n**  **(min-max)** | **age** | **T (°C)** | **Kidney pathology (%)** | **Tb positive real-time PCR (%)** | **Days/year >15°C in SY**  **(CHMI)** | **Days to catch >15°C in SY**  **(CHMI)** |
| --- | --- | --- | --- | --- | --- | --- | --- | --- | --- | --- |
| 16.9. 2020 | ^1^Opava 2 | 50°4'20.689"N 17°27'31.630"E | 16 | 143-173/16 | 1+ | 11.6 | 0 | 12.5 | n.a. | n.a. |
| 6.8. 2024 | ^1^Opava 3 | 50°5'10.361"N 17°41'34.953"E | 10 | 162 and 177/2  180-226/8 | 1+  2+ | 15.6 | 0 | 90.0 | 140 | 85 |
| 25.7. 2020 | ^1^Moravice | 49°51'15.463"N 17°50'53.597"E | 16 | 153-172/16 | 1+ | 15.9 | 0 | 56.2 | 56 | 23 |
| 1.11. 2020 | ^1^Ostravice | 49°36'32.834"N  18°21'27.166"E | 16 | 135-170/16 | 1+ | 10.7 | 0 | 12.5 | n.a. | n.a. |
| 6.8. 2024 | ^1^Lomná | 49°34'22.839"N 18°45'27.993"E | 10 | 176-180/3  191-219/7 | 1+  2+ | 20.0 | 0 | 0 | n.a. | n.a. |
| 6.8. 2024 | ^1^Lučina | 49°44'14.846"N  18°26'27.503"E | 2 | 235 and 262 | 3+ | 13.6 | 0 | 100 | 38 | 0 |
| 11.9. 2020 | ^2^Vsetínská Bečva | 49°21'8.939"N 18°13'54.089"E | 17 | 92-123/9  135-137/2  184-204/6 | 0+  1+  2+ | 12.3 | 0 | 0 | n.a. | n.a. |
| 7.8. 2024 | ^2^Vsetínská Bečva | 49°18'41.845"N 18°0'14.482"E | 5 | 216 and 225  259-285/3 | 2+  3+ | 21.2 | 0 | 40 | n.a. | n.a. |
| 7.8. 2024 | ^2^Rožnovská Bečva | 49°25'39.288"N 18°17'41.378"E | 10 | 170-181/5  194-209/5 | 1+  2+ | 14.8 | 0 | 70 | 89 | 51 |
| 7.8. 2024 | ^2^Rožnovská Bečva | 49°28'10.379"N 17°57'25.623"E | 5 | 206-238/3  252 and 261 | 2+  3+ | 23.6 | 0 | 40 | 118 | 81 |
| 5.8. 2024 | ^2^Morava | 50°5'25.137"N  16°53'42.826"E | 10 | 137-172/5  192-209/5 | 1+  2+ | 14.4 | 0 | 0 | 0 | 0 |
| 5.8. 2024 | ^2^Morava | 50°1'40.265"N 16°53'59.178"E | 10 | 160-171/3  181-219/5  241 and 255 | 1+  2+  3+ | 15.0 | 0 | 0 | 64 | 33 |
| 3.9. 2024 | ^2^Bystřice | 49°35'52.437"N 17°21'49.751"E | 8 | 185 and 224  254-263/6 | 2+  3+ | 19.1 | 0 | 37.5 | n.a. | n.a. |
| 5.8. 2024 | ^2^Desná | 50°5'59.057"N  17°7'0.962"E | 10 | 151-179/3  185-197/5  234 and 256 | 1+  2+  3+ | 16.7 | 0 | 0 | 60 | 32 |
| 5.8. 2024 | ^2^Desná | 49°57'11.171"N  16°59'23.618"E | 10 | 165-173/3  180-228/6  243 | 1+  2+  3+ | 16.6 | 0 | 100 | n.a. | n.a. |
| 18.9. 2020 | ^3^Březná | 49°57'32.519"N 16°45'56.956"E | 17 | 100-128/17 | 0+ | 10.9 | 100 | 100 | n.a. | n.a. |
| 19.7. 2023 | ^3^Chvalšinský potok | 48°49'1.428"N  14°17'23.703"E | 9 | 175-180/4  191-203/5 | 1+  2+ | 15.5 | 22 | 100 | 76 | 30 |
| 19.7. 2023 | ^3^Chvalšinský potok | 48°49'30.130"N  14°15'16.852"E | 10 | 73-82/5  176  195-219/4 | 0+  1+  2+ | 15.3 | 0 | 70 | n.a. | n.a. |
| 19.7. 2023 | ^3^Polečnice | 48°47'22.374"N  14°14'25.161"E | 10 | 170-216/10 | 2+ | 15.3 | 0 | 100 | n.a. | n.a. |
| 19.7. 2023 | ^3^Křemžský potok | 48°55'44.821"N  14°12'44.812"E | 10 | 156-183/10 | 1+ | 15.4 | 0 | 90 | n.a. | n.a. |
| 19.7. 2023 | ^3^Křemžský potok | 48°53'40.881"N 14°19'49.846"E | 10 | 184-219/10 | 2+ | 15.3 | 10 | 100 | n.a. | n.a. |
| 20.7. 2023 | ^3^Malše | 48°42'37.344"N 14°29'14.014"E | 10 | 150-182/7  190-200/3 | 1+  2+ | 20.6 | 20 | 90 | n.a. | n.a. |
| 20.7. 2023 | ^3^Malše | 48°44'31.716"N 14°29'28.936"E | 9 | 168-180/6  190-220/3 | 1+  2+ | 19.9 | 44 | 100 | n.a. | n.a. |
| 20.7. 2023 | ^3^Malše | 48°45'16.296"N 14°30'35.477"E | 10 | 155-180/5  193 and 215  235-258/3 | 1+  2+  3+ | 20.6 | 0 | 100 | 106 | 47 |
| 20.7. 2023 | ^3^Černá | 48°43'48.174"N  14°34'18.307"E | 10 | 149-176/10 | 1+ | 19.0 | 0 | 20 | 54 | 24 |
| 21.7. 2023 | ^3^Teplá Vltava | 48°57'15.374"N  13°45'23.025"E | 10 | 143-167/9  185 | 1+  2+ | 13.9 | 0 | 0 | n.a. | n.a. |
| 21.7. 2023 | ^3^Teplá Vltava | 48°56'16.110"N 13°47'35.783"E | 10 | 127-150/10 | 1+ | 15.0 | 0 | 0 | 39 | 18 |
| 21.7. 2023 | ^3^Blanice | 48°59'51.936"N 13°55'14.403"E | 10 | 140-155/8  178 and 186 | 1+  2+ | 15.3 | 0 | 10 | n.a. | n.a. |
| 21.7. 2023 | ^3^Blanice | 49°1'30.677"N  13°56'40.882"E | 10 | 134-172/6  180-186/4 | 1+  2+ | 17.5 | 0 | 0 | 67 | 32 |
| 25.7. 2023 | ^3^Volyňka | 49°5'35.674"N  13°47'42.504"E | 11 | 146-168/9  187/1 | 1+  2+ | 16.2 | 0 | 0 | n.a. | n.a. |
| 25.7. 2023 | ^3^Volyňka | 49°13'59.082"N 13°53'35.521"E | 10 | 155-185/6  193-205/4 | 1+  2+ | 17.5 | 0 | 70 | 104 | 45 |
| 26.7. 2023 | ^3^Novosedelský potok | 49°15'39.970"N  13°47'51.972"E | 10 | 188-222/10 | 2+ | 15.3 | 10 | 100 | n.a. | n.a. |
| 26.7. 2023 | ^3^Spůlka | 49°6'47.195"N  13°43'5.151"E | 10 | 148-175/9  189/1 | 1+  2+ | 15.0 | 0 | 80 | n.a. | n.a. |
| 26.7. 2023 | ^3^Spůlka | 49°6'19.898"N  13°46'46.533"E | 10 | 133-172/5  180-190/5 | 1+  2+ | 14.1 | 0 | 50 | 65 | 36 |
| 26.7. 2023 | ^3^Stropnice | 48°45'6.498"N  14°44'35.796"E | 10 | 140-177/10 | 1+ | 13.6 | 0 | 20 | 67 | 36 |
| 26.7. 2023 | ^3^Svinenský potok | 48°46'45.780"N, 14°40'27.605"E | 10 | 155-170/5  180-205/5 | 1+  2+ | 15.6 | 0 | 90 | n.a. | n.a. |
| 9.8. 2023 | ^3^Kosový potok | 49°52'16.788"N  12°48'22.832"E | 5 | 192-202/4  253/1 | 2+  3+ | 15.8 | 0 | 100 | n.a. | n.a. |
| 9.8. 2023 | ^3^Střela | 50°5'42.897"N  13°16'57.551"E | 10 | 146-178/6  183-203/4 | 1+  2+ | 15.2 | 0 | 100 | 46 | 30 |
| 9.8. 2023 | ^3^Ohře | 50°21'30.306"N  13°28'12.401"E | 8 | 205-244/7  257/1 | 2+  3+ | 13.7 | 0 | 0 | n.a. | n.a. |
| 10.8. 2023 | ^3^Chřibská Kamenice | 50°50'15.982"N  14°22'27.542"E | 10 | 163-181/5  186-216/5 | 1+  2+ | 14.3 | 0 | 100 | n.a. | n.a. |
| 10.8. 2023 | ^3^Kamenice | 50°49'3.138"N  14°21'6.092"E | 9 | 194-224/7  234-260/3 | 2+  3+ | 14.0 | 55 | 100 | n.a. | n.a. |
| 10.8. 2023 | ^3^Kamenice | 50°52'30.972"N  14°14'51.129"E | 10 | 148-183/7  203-222/3 | 1+  2+ | 19.7 | 0 | 100 | 0 | 0 |
| 21.9. 2023 | ^3^Jizerka | 50°49'7.040"N 15°20'53.798"E | 10 | 116-131/3  151-173/5  182 and 190 | 1+  2+  3+ | 9.7 | 0 | 100 | 0 | 0 |
| 21.9. 2023 | ^3^Jizera | 50°42'23.243"N 15°25'26.357"E | 10 | 118-166/10 | 1+ | 13.4 | 0 | 0 | 41 | 41 |
| 21.9. 2023 | ^3^Elbe | 50°42'35.798"N 15°35'5.709"E | 10 | 145-171/8  183 and 213 | 1+  2+ | 15.6 | 0 | 0 | n.a. | n.a. |
| 21.9. 2023 | ^3^Elbe | 50°33'54.248"N  15°39'4.600"E | 10 | 145-175/8  184 and 191 | 1+  2+ | 17.0 | 0 | 100 | 86 | 85 |
| 21.9. 2023 | ^3^Metuje | 50°31'45.087"N  16°11'23.826"E | 10 | 159-178/5  181-220/5 | 1+  2+ | 13.6 | 20 | 100 | n.a. | n.a. |
| 21.9. 2023 | ^3^Metuje | 50°21'5.479"N  16°7'31.555"E | 6 | 214-241/4  254 and 287 | 2+  3+ | 16.1 | 0 | 100 | n.a. | n.a. |
| 21.9. 2023 | ^3^Divoká Orlice | 50°16'49.679"N 16°28'31.618"E | 10 | 133-136/6  179-194/4 | 1+  2+ | 13.5 | 0 | 0 | n.a. | n.a. |
| 21.9. 2023 | ^3^Tichá Orlice | 50°5'51.038"N 16°40'24.358"E | 4 | 202-246/4 | 2+ | 16.2 | 25 | 100 | n.a. | n.a. |
| 21.9. 2023 | ^3^Tichá Orlice | 49°59'8.774"N 16°23'27.562"E | 8 | 182-226/8 | 2+ | 12.7 | 25 | 100 | 25 | 25 |
